# *Viscum album* (mistletoe) extract for dogs with cancer?

**DOI:** 10.3389/fvets.2023.1285354

**Published:** 2024-01-03

**Authors:** Hans Klingemann

**Affiliations:** No Longer Running Behind Foundation, Boston, MA, United States

**Keywords:** dogs, cancer therapy, mistletoe, *Viscum album*, immunotherapy

## Abstract

Compared with the options available to human patients with cancer, treatment choices for dogs are often more limited. Chemotherapy is frequently the first-line treatment for many cancers. However, its efficacy can be limited, and its side effects can affect the quality of the remaining life. This paper briefly summarizes the experience with *Viscum album L.* (mistletoe) extract in human patients as a stipulation to consider treatment with mistletoe extract for canines with cancer. The mistletoe extract contains -among others - lectins and viscotoxins that have documented anti-proliferative effect on cancer cells as well as immune-stimulatory function. Importantly, it also improves the well-being of patients with cancer due to its lectin ML-1 content, which can trigger the release of endorphins. Being cross-reactive with canine cells and having a relatively low side effect profile, it raises the question of whether mistletoe preparations might be considered as part of the treatment approach for dogs with cancer.

## 1 Introduction

### 1.1 What is mistletoe?

**Figure 1:**
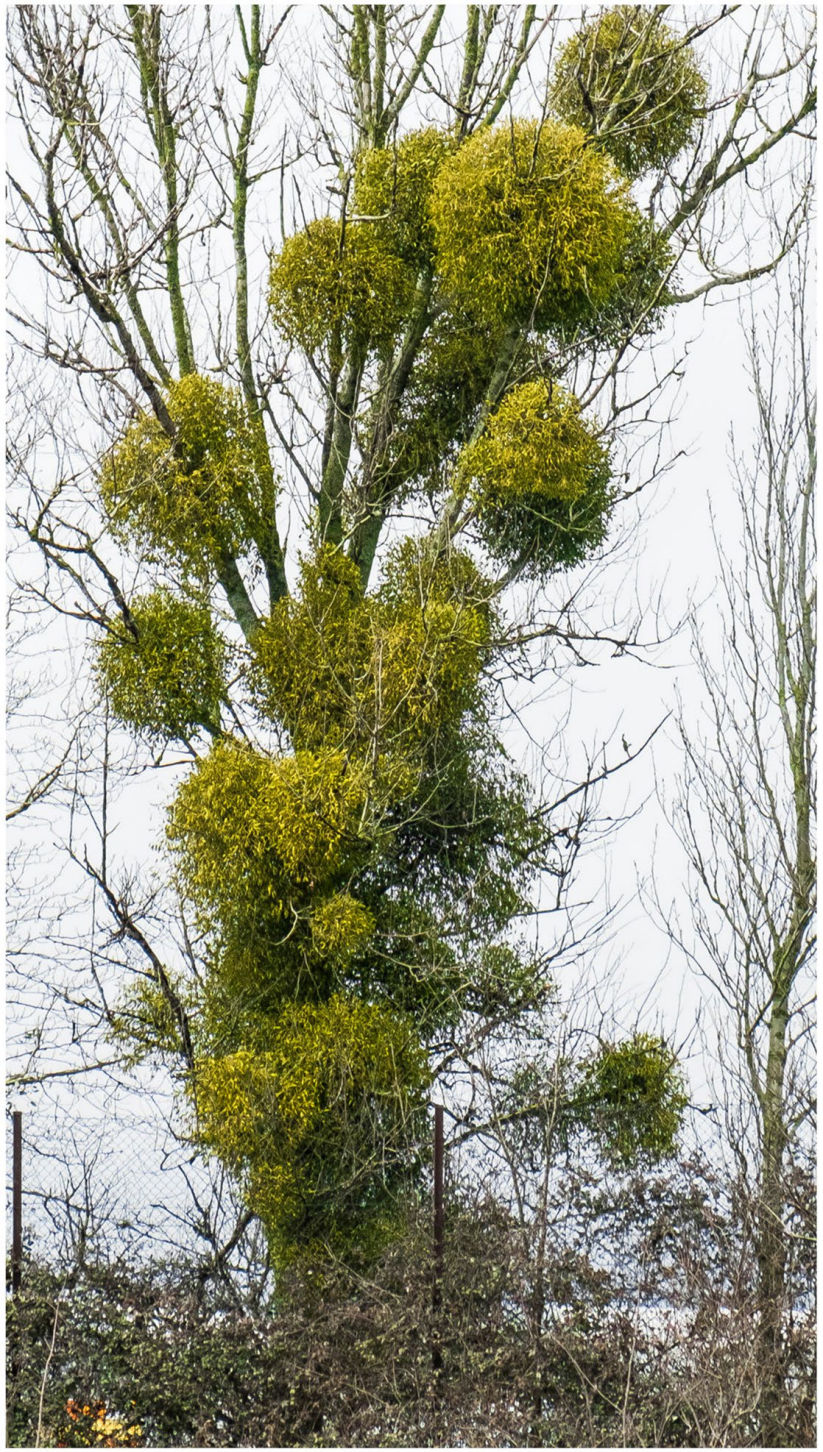
Mistletoe growing on host tree.

It is a semi-parasitic plant that grows on trees and uses their sap to thrive (Figure 1). In fact the trees it grows on, often die off over time. Mistletoe has been around as a cancer therapeutic for more than a century especially in the German speaking part of Europe (1). It was introduced by Rudolf Steiner and Dr. Ita Wegman, who treated the first patients with cancer with *Viscum album* mistletoe extract around 1920. In addition to some tumor responses, it was also noted throughout the years that the quality of life of patients with cancer even during chemo/radiotherapy, could be improved with mistletoe (2–4). In fact, mistletoe extracts are currently the most frequently prescribed non-conventional cancer treatment in central European countries; 70–80% of patients with cancer will receive it at some point.

Most bioactive ingredients of mistletoe have been identified and characterized such as lectins (I, II, and III), polypeptides (e.g., viscotoxins), and immunostimulatory glycoproteins (5–7). The extracts are also enriched in biologically active flavonoids, phenolic acids, sterols, lignans, terpenoids, and phenylpropanoids (8). There is strong evidence that the *complete* mistletoe extract is more potent than when isolated compounds are administered (9, 10). Mistletoe preparations are made from extracts of leaves, stems, buds, and ripe berries during the fall or winter harvest (11). Although some 1,500 species of plants are denoted as mistletoes, only white-berry mistletoe from Northern Europe (*Viscum album* L.) is used in cancer treatment. The tree *origin* of the mistletoe is also relevant (pine, apple, oak, or ash) and so is the *time of harvest* (fall vs. winter) as the concentration of the different active components varies with the time of the year (12). For example, green berries in the fall have more viscotoxins, whereas white berries in winter carry more lectins (13). The extract can also be fermented, a process that can add bacterial metabolites to the extract, which can function as pathogen-associated molecular patterns [PAMP] (14). *Iscador^R^* is fermented, whereas *Helixor^R^* and *abnobaViscum^R^* are not.

Numerous *in vitro* studies have confirmed the direct inhibitory effect of mistletoe on malignant cell proliferation and apoptosis (15–17). Researchers from MD Anderson Cancer Center determined in a liver cancer model that this effect is related to certain components in mistletoe that downregulate the expression of the c-myc oncogene in cancer cells (18). In addition to anti-proliferative /apoptotic effects, mistletoe stimulates the secretion of immune-active cytokines (19) and augments the function of immune cells, such as T-lymphocytes (20, 21) and natural killer cells (22, 23). It also supports the maturation of dendritic cells and macrophages (24, 25). Mistletoe ingredients have an anti-angiogenic effects in cancer tissues and neutralize tumor-induced immunosuppression (17). Importantly, mistletoe can improve the quality of life of cancer patient (2–4). Even in the advanced stages, it mitigates cancer-related symptoms and reduces the side effects of chemotherapy and radiation. This beneficial effect is related to the lectin ML-1 content, which stimulates the release of endorphins (26).

Despite the widespread use of mistletoe preparations in central Europe, it has not found its place in the US and Canada as the FDA has not granted its stamp of approval largely due to the fact that the commercial mistletoe extracts have multiple ingredients which makes it difficult to standardize each batch for a given ingredient. However, the production of mistletoe follows a standardized manufacturing process and its batch to batch consistent biological activity is guaranteed.

Although there are numerous reports that mistletoe extracts have a therapeutic benefit in cancer patients in terms of response rate, overall survival, and quality of life, many of these studies have a major challenge: when mistletoe is administered concurrently with chemotherapy or radiation, it becomes difficult to define its contribution. Since the use of mistletoe is so prevalent in Germany, Troger et al. (27) went outside of the country to perform a randomized trial with mistletoe in patients with locally advanced pancreas cancer. Patients in the mistletoe arm had a significantly higher tumor response rate and longer survival time than those in the control group who received standard chemotherapy. In another study, mistletoe extract was given to 23 patients with advanced, chemotherapy naïve liver cancer with five patients (22%) achieving a complete or partial response (28). Acute myeloid leukemia is also responsive to *Viscum album* as reported from *in vitro* and *in vivo* (murine) studies (29). It is noteworthy that mistletoe has quite limited side effects when administered at the recommended dose, and the side effects that have been reported more frequently (inflammatory reaction at the injection site, fever, malaise) are the ones that physicians want to see with any immunotherapy as signs of an active immune response.

Owing to the initiative of a patient who had received mistletoe treatment for metastatic cancer, a Foundation (*“Believe Big”* https://www.believebig.org/what-is-mistletoe/) was initiated with the goal of supporting the clinical exploration of mistletoe in the US in a well-controlled clinical study setting. With funding from that initiative, a phase I study was conducted at Johns Hopkins in 21 patients with various advanced cancers who received mistletoe intravenously (600 mg) thrice a week until disease progression or toxicity occurred. The results of this safety/feasibility study were recently published (30). Side effects were minor, and 25% of the patients were reported to have stable disease with a median follow-up of 15.3 months. A reduction in tumor size occurred in three patients remaining stable for 2–5 months. Importantly, most patients reported an improvement in their quality of life. Further clinical trials are planned to determine the efficacy of mistletoe in different cancer sites.

For human patients with cancer, mistletoe (whole plant extracts) is usually administered *intravenously* at the beginning of the treatment cycle, followed by *subcutaneous* administration (i.e., 3 times a week). Intra-tumor application is particularly attractive in early stage cancer (even during or after surgery) when the tumor has not yet spread to other organs. Intra-tumor injection of ash tree- derived mistletoe into a human pancreatic cancer xenograft resulted in significant tumor response, with 75% of treated mice having either a partial or complete response (31). In a safety study, Steele et al. (32) treated 123 patients with cancer with intra-tumor mistletoe injections from various providers. The side effects were relatively mild, and consisted only of fever and local inflammatory reactions. In this context, a promising indication for local mistletoe administration is bladder cancer (33–35). It is important to note that mistletoe supplements in the form of capsules, liquid extracts, teas, and powders have no scientific or clinical support for efficacy.

### 1.2 Rationale for using mistletoe for dogs with cancer

Despite the evidence of an anti-cancer effect in humans and its ability to improve the quality of life of patients with cancer, there is very limited well-documented experience with *Viscum album* in treating cancer in dogs. There may also be a perception that the berries are poisonous to dogs (they contain Viscumin). However, dogs have to eat a fair amount and even after accidental ingestion, signs and symptoms are limited (36). There is also a difference between uncontrolled and accidental ingestion of the plants/berries and administration of a medicinal preparation, which is well defined and prepared in pharmacological doses. Kienle et al. (36) reviewed the safety of various mistletoe preparations and doses in animals (mostly mice, one horse, one cat, no dogs) and noted minimal or only low grade side effects even at higher doses.

Although surgery, radiation, and chemotherapy are considered first-line treatments for most canine patients with cancer, there are many scenarios in which this approach fails, or the dog cannot tolerate it at some point. Not infrequently, owners cannot see the dog suffering from the side effects of chemotherapy, and the quality of life becomes a consideration. In fact, in a recent survey, it was found that about two-third of dog owners would not elect to treat their dog with chemotherapy due to the negative impact of the associated side effects (37).

Although Immunotherapy has become the fourth pillar of cancer therapy for humans, it is far less developed for dogs (38, 39). Considering its beneficial reports in human patients with cancer, it is surprising that *Viscum album* extracts have not received more attention as immune-active cancer treatment for our “best friend.” The United States Department of Agriculture [USDA] regulates drug use in the veterinary space, and as long as mistletoe is not officially licensed, reimbursement will be limited. In fact, since all the companies that produce clinical-grade mistletoe are located in Europe, imports into the US are also regulated. However, there are some veterinarians in the US who can provide mistletoe treatment despite limited access and logistical challenges.

### 1.3 Current status of mistletoe use for cancer treatment in dogs

A literature search for studies on canine cancer cell lines exposed to mistletoe resulted in only one study that confirmed its cytotoxicity against canine astrocytoma cells (40). There have been less than a handful of clinical studies exploring the use of mistletoe in dogs. Biegel et al. (41) treated dogs with mammary tumors with mistletoe subcutaneously in the adjuvant setting after surgery. Compared to the non-treated control group, there was a trend (*p* = 0.07) toward a decrease in tumor-related death while maintaining a stable quality of life for a prolonged time. The same investigators treated dogs with oral malignant melanoma with mistletoe after radiation in a non-randomized study (42, 43). Eighteen dogs received mistletoe subcutaneously, while eight did not. The median survival time in the treatment group was 236 days versus 49 days in dogs that did not receive mistletoe.

Where to go from here? To convince veterinarians that mistletoe can have some benefits for dogs with cancer, the first step would be to conduct some comprehensive *in vitro* studies with canine cancer cell lines and tumor biopsy material to define which canine tumors are more sensitive to the cytotoxic and immunomodulatory effects of mistletoe. The next step would be to conduct phase I studies that would test the safety of escalating subcutaneous injections in dogs using the three times/week schedule adopted from humans. Ideally some pharmacokinetic studies can be included to assure that the dosage derived from human administration applies equally to dogs. Although mistletoe preparation have many components, it appears that the lectin plasma level can be reliably measured (44). With this knowledge, clinical trials could determine in which diseases mistletoe is most effective for dogs with cancer even as an alternative in situations where owners decide against more aggressive treatment. Despite the challenges of obtaining funding for veterinary trials, it would be relevant to look at the effect of mistletoe administered intra-tumor or locally, such as in melanoma and bladder cancer, if the tumor is accessible and has not metastasized. It may be more challenging to quantify the effect of the treatment on improving the quality of life of canine patients, as there are fewer well-established parameters in place (45). Considering the available facts though, *Viscum album*/mistletoe is a treatment option that should not be withheld for dogs considering the unequivocal benefits reported in human patients with cancer for more than a century.

## Author contributions

HK: Conceptualization, Writing - original draft, Writing - review and editing.
